# The Influence of *Spirulina platensis* Filtrates on Caco-2 Proliferative Activity and Expression of Apoptosis-Related microRNAs and mRNA

**DOI:** 10.3390/md15030065

**Published:** 2017-03-07

**Authors:** Agnieszka Śmieszek, Ewa Giezek, Martyna Chrapiec, Martyna Murat, Aleksandra Mucha, Izabela Michalak, Krzysztof Marycz

**Affiliations:** 1 Department of Environment Hygiene and Animal Welfare, The Faculty of Biology and Animal Science, Wroclaw University of Environmental and Life Sciences, 38 C Chelmonskiego St., 50-630 Wroclaw, Poland; ewa.giezek@gmail.com (E.G.); m.chrapiec@gmail.com (M.C.); martyna.murat@wp.pl (M.M.); olka.mucha@gmail.com (A.M.); krzysztof.marycz@up.wroc.pl (K.M.); 2 Electron Microscopy Laboratory, The Faculty of Biology and Animal Science, University of Environmental and Life Sciences, Kożuchowska 5b Street, 50-631 Wroclaw, Poland; 3 Department of Advanced Material Technologies, Faculty of Chemistry, Wrocław University of Science and Technology, 54-066 Wrocław, Poland; izabela.michalak@pwr.edu.pl; 4 Wroclaw Research Centre EIT+, Stablowicka 147, 54-066 Wroclaw, Poland

**Keywords:** *Spirulina*, colon, human colon cancer, cytotoxic effect, anti-cancer effect, proliferation, morphology, apoptosis

## Abstract

*Spirulina platensis* (SP) is a blue-green microalga that has recently raised attention not only as a nutritional component, but also as a source of bioactivities that have therapeutic effects and may find application in medicine, including cancer treatment. In the present study we determined the cytotoxic effect of *S. platensis* filtrates (SPF) on human colon cancer cell line Caco-2. Three concentrations of SPF were tested—1.25%, 2.5%, and 5% (*v*/*v*). We have found that the highest concentration of SPF exerts the strongest anti-proliferative and pro-apoptotic effect on Caco-2 cultures. The SPF negatively affected the morphology of Caco-2 causing colony shrinking and significant inhibition of metabolic and proliferative activity of cells. The wound-healing assay showed that the SPF impaired migratory capabilities of Caco-2. This observation was consistent with lowered mRNA levels for metalloproteinases. Furthermore, SPF decreased the transcript level of pro-survival genes (cyclin D1, surviving, and c-Myc) and reduced the autocrine secretion of Wnt-10b. The cytotoxic effect of SPF involved the modulation of the Bax and Bcl-2 ratio and a decrease of mitochondrial activity, and was related with increased levels of intracellular reactive oxygen species (ROS) and nitric oxide (NO). Moreover, the SPF also caused an increased number of cells in the apoptotic sub-G0 phase and up-regulated expression of mir-145, simultaneously decreasing expression of mir-17 and 146. Obtained results indicate that SPF can be considered as an agent with anti-cancer properties that may be used for colon cancer prevention and treatment.

## 1. Introduction

Micro- and macro-algal extracts have become important sources of bioactive molecules with anti-cancer properties [1,2,3,4]. Their cytotoxic effect was established for various cancer cell lines, for example, human hepatocarcinoma cell line (HepG2) [5], human breast carcinoma cell line (MCF-7) [6], but also human colon carcinoma cell lines, including Caco-2 [7,8,9]. The wide range of health-promoting effects described for algae includes, for example: (i) controlling of blood glucose levels and improving lipid profiles in patients with type 2 diabetes [7,8]; (ii) antiviral activity, like inhibition of HIV replication [9]; (iii) positive influence on nutritional status of HIV-infected children [10] and (iv) improvement of lung function of patients with asthma [11]. This contributes to the great possibilities in potential pharmacological application of algal bioactives.

The most widely studied alga, in the context of high anti-oxidative properties, is *Spirulina platensis*, which is a free-floating filamentous blue-green microalga belonging to the class of cyanobacteria [12,13]. Despite the fact that *Spirulina* was a crucial element of ancient diets, its popularity has emerged again relatively recently. Currently, *Spirulina* is considered as a functional food and diet supplementation [14].

Nutritional value provided by *Spirulina platensis* depends mainly on its high content of proteins, amino acids, and polyunsaturated fatty acids, including gamma-linoleic acids, but also vitamins and minerals. Chlorophyll, β-carotene, and lutein produced by the microalgae are the guarantors of their powerful antioxidant properties [12,15]. Additionally, it was shown that *Spirulina* has higher content of phenolic compounds, when compared to *Chlorella*—another alga popular in nutrition [16].

The inhibition of cancerogenesis induced by biologically active compounds derived from *Spirulina* was tested both in vitro and in vivo [11]. Obtained data strongly linked anti-cancer effects of *S. platensis* to its antioxidant activity. The in vitro studies showed that cytotoxic effect of *Spirulina* involved a decrease of proliferative capacity of tumorigenic cell lines that could result from mitochondrial activity disturbance [17]. Additionally, Ismail et al. showed that *S. platensis* may induce apoptosis of the hepatocellular carcinoma cell line HepG2, increasing the Bax/Bcl-2 ratio [11]. The survival and proliferation of cancer cells is controlled by small non-coding RNAs—micro RNAs (miRNAs). For example, in vitro studies showed that suppression of miR-145 may inhibit proliferation, motility, and invasion of colorectal cancer cell lines (SW620 and LoVo) [18]. The profile of expression miRNAs in colon tissue may also have diagnostic value—the overexpression of miR-17 and downregulation of miR-146 was associated with colon cancer progression [19,20].

*Spirulina* was also proved to have anti-precarcinogenic potential in vivo—it significantly reduced the number of aberrant crypts formed in the colon. This dependency was shown using rat [21] and mice [22] in an in vivo model.

The aim of the present study was to evaluate potential anti-cancer activity of *S. platensis* water extracts on the Caco-2 cell line. We determined the influence of SPF on proliferation, morphology, and transcript levels of genes strongly related to apoptosis. In our field of interest was also the effect of SPF on miR17, miR145, and miR146 expression profiles. Moreover, due to the fact that mitochondria damage is often observed in cells subjected to anti-cancer treatment, we also investigated the SP influence on mitochondria status and reactive oxygen species generation in Caco-2 cultures.

## 2. Materials and Methods

### 2.1. Cell Line

The Caco-2 cells, originating from human colon cancer, were obtained from the American Type Culture Collection (ATCC) at passage 43 (ATCC^®^ HTB-37™). Caco-2 cells were maintained and expanded in a 75 cm^2^ flask at 37 °C in an incubator with 95% humidity and 5% CO_2_. The growth medium—Dulbecco’s Modified Eagle’s Medium (DMEM, Sigma Aldrich, Munich, Germany) contained 4500 mg/L of glucose and was supplemented with 10% of fetal bovine serum (FBS), 1% l-glutamine solution (100× BioWest S.A.S, Nuaillé, France), 1% MEM solution (100×, Gibco^®^ Thermo Fisher Scientific, Warszawa, Poland), 2% HEPES buffer (1 M, Gibco^®^ Thermo Fisher Scientific, Warszawa, Poland), and 1% antibiotic-antimycotic solution (Sigma Aldrich, Munich, Germany). Complete growth medium (CGM) was changed every two days. Passage was performed when cells reached about 80%–90% of confluence, and the procedure was performed using trypsin solution (TrypLE™ Express; Thermo Fisher Scientific, Warszawa, Poland) accordingly to the instructions provided by manufacturer.

### 2.2. Preparation of Spirulina platensis Filtrate (SPF)

The *Spirulina* was derived from Mühle Ebert Dielheim GmbH (MED, Dielheim, Germany). One day before the experiments *S. platensis* powder (10 mg) was soaked in complete culture medium (1 mL) with the 2% addition of antibiotic-antimycotic solution. The mixture was shaken continuously and incubated overnight at 37 °C. The obtained supernatant was collected and filtrated using syringe filters, first with a 0.45 µm pore size, then using filters with a 0.22 µm pore size. The obtained filtrate was maintained at 4 °C.

### 2.3. The Experiment

The Caco-2 cell line (passage 50) was inoculated in 0.5 mL of CGM into 24-well plates at concentration equal to 3 × 10^6^ cells per well. The SPF was added to the Caco-2 cultures at final concentrations of 1.25%, 2.5%, and 5% (*v*/*v*). The control of the experiment consisted of Caco-2 cultures without the SPF addition. Each assay was performed in three replicates. The cultures were propagated for 72 h, and the medium was changed every day.

### 2.4. Analysis of Caco-2 Proliferation in Cultures with SPF

#### 2.4.1. Metabolic Assay (Alamar Blue)

Cell viability was investigated using resazurin-resorufin system (*tox-8*, Sigma Aldrich, Munich, Germany). Metabolic activity of cells was evaluated after 24 and 72 h of culture. The test was performed in accordance with the manufacturer’s instructions and based on protocols described in detail previously [23]. The relative values expressed metabolic activity of Caco-2 in cultures with SPF in relation to the control culture.

#### 2.4.2. Analysis of DNA Synthesis (BrdU Incorporation Assay)

The proliferation of Caco-2 in cultures with SPF was quantified using a bromodeoxyuridine (BrdU) ELISA Kit (Abcam, Cambridge, UK). For this purpose cultures were maintained in a 96-well plate. The analysis were performed after 24 and 72 h of cell culture. Testing was performed according to the manufacturer’s instructions. The reaction was read using a spectrophotometer microplate reader (Spectrostar Nano, BMG Labtech, Ortenberg, Germany) at a wavelength of 450/550 nm.

#### 2.4.3. Determination of Colony Forming Efficiency (CFE)

Determination for colony formation was assessed by seeding the Caco-2 (p. 50) in cultures with SPF into six-well plate. The cells were cultured in CGM and the cell density was equal to 100 cells per well. Next, the cells were maintained for 10 days in a CO_2_ incubator (5% CO_2_ and 95% humidity). The medium was removed and cells were fixed with 4% paraformaldehyde (PFA) and stained with pararosaniline (Sigma Aldrich) for 10 min at room temperature. The CFU were counted as colonies consisting only of 50 or more cells according to the method described previously [24]. The experiment was performed three times.

### 2.5. Oxidative Stress Factors in Caco-2 Cultures with SPF

The oxidative stress factors were determined in supernatants collected after 72 h of Caco-2 culture. The intracellular reactive oxygen species (ROS) were measured using H2DCF-DA solution (Thermo Fisher Scientific, Warszawa, Poland), while superoxide dismutase (SOD) was assessed using a commercially available SOD determination kit. Nitric oxide (NO) activity was assessed using a Griess reagent kit (Thermo Fisher Scientific, Warszawa, Poland). The assays were performed in three replicates. The absorbance of the supernatants was measured spectrophotometrically at a wavelength of 560 nm and 450 nm, respectively, using a microplate reader (BMG LABTECH, Ortenberg, Germany). ROS, SOD, and NO analysis were performed according to the manufactures’ protocol.

### 2.6. Morphology of Caco-2 in Cultures with SPF

#### 2.6.1. Inverted and Epifluoresence Microscope

Cultures after 24 and 72 h of propagation were observed under an epifluorescence microscope (Axio Observer A.1; Zeiss, Oberkochen, Germany) and captured using a PowerShot camera (Canon, Warszawa, Poland). Afterward, experimental cultures were fixed with 4% paraformaldehyde and additionally stained using diamidino-2-phenylindole (DAPI; 1:1000) and atto-565-labeled phalloidin (1:800). Both DAPI and phalloidin derived from Sigma Aldrich (Munich, Germany). A detailed description of staining procedure was described previously [25].

#### 2.6.2. Scanning Electron Microscope (SEM)

For visualization in SEM, cultures were fixed with 4% paraformaldehyde and dehydrated with graded series of ethanol (from 50% to 100%, every 10%), each incubation lasted 5 min. Dry preparations were coated with gold particles using a 300-s program (Edwards, Scancoat six). Cultures were analyzed using an SE1 detector with a 10 kV filament tension (SEM, Evo LS 15; Zeiss, Oberkochen, Germany) and 1000× magnification.

### 2.7. Wound Healing Assay

In order to perform the wound healing test, the Caco-2 cultures (p. 50) were maintained in 24-well plates, enforcing high confluency. Cell monolayers were manually wounded by scraping cultures with yellow pipette tips (0.5–0.6 mm diameter). After that, cultures were washed twice to remove the detached cells and propagated with SPF in a CO_2_ incubator for 24 h. Furthermore, cultures were stained with pararosaniline (Sigma Aldrich, Munich, Germany). Determination of wound closure was performed using AxioVision microscope software. The measurements of distance, obtained between the two edges of a denuded area, were performed five times and repeated independently in triplicate.

### 2.8. Visualization of Mitochondria

In order to visualize the mitochondria, cells were incubated with Mito Red dye (1:1000) in 37 °C for 30 min in a CO_2_ incubator. Following fixation with PFA, cells were rinsed three times with HBSS. The cultures were then observed under an epifluorescence microscope (Axio Observer A.1 Zeiss, Oberkochen, Germany) and captured using a PowerShot camera (Canon, Warszawa, Poland). The intensity of fluorescence signal derived from mitochondrial was analyzed using ImageJ software with a Pixel Counter plugin (National Institutes of Health, Bethesda, MD, USA, http://imagej.nih.gov/ij/).

### 2.9. Live/Dead Staining

The viability of Caco-2 in cultures with SPF was investigated using a two-color fluorescence live/dead assay (Double Staining Kit: Sigma Aldrich, Munich, Germany). The staining was performed according to the manufacture’s protocol. The experiment was conducted in triplicate. The observation of stained cultures was performed with epifluorescence microscope (Axio Observer A.1) and captured using a PowerShot camera (Canon, Warszawa, Poland). Determination of dead cell percentage was done using ImageJ software (National Institutes of Health, Bethesda, MD, USA, http://imagej.nih.gov/ij/).

### 2.10. Analysis of Cell Cycle

The Caco-2 (p. 50) were cultured with SPF at defined concentrations in T-25 dishes after 72 h. After the experiment, cultures were trypsinized, and pellets containing 2 × 10^6^ cells were fixed with ice-cold 70% ethanol, added in a drop-wise manner while gently vortexing. Cells were kept for one week at 4 °C. After fixation stage, the cells were centrifuged at 800× *g* for 5 min, and washed with HBSS three times. Pellets were resuspended in HBSS containing 50 µg/mL propidium iodide and 100 µg/mL of RNase A. Samples were incubated for 30 min at 37 °C, protected from light. Analysis and measurement of propidium iodide fluorescence were performed on a FACSCalibur (BD Biosciences, Franklin Lakes, NJ, USA) flow cytometer. For each analysis 20,000 events were acquired. The data were analyzed using FlowJo X software (Treestar, Ashland, OR, USA, trial version 2016).

### 2.11. Analysis of Wnt-10b Concentration in Supernatants after Caco-2 Culture with SPF

The concentration of secreted Wnt-10b was determined in supernatants collected after experimental cultures of Caco-2 with and without *S. platenis* filtrate. Analysis was performed using specific enzyme-linked immunosorbent assays derived from Wuhan EIAab Science Co., Ltd. (Wuhan, China), characterized by the detection range 0.312–20 ng/mL. For the analysis supernatants were undiluted. All tested samples and standards were measured in triplicate. Optical density was determined immediately after reactions and measured at 450 nm using a microplate reader (Spectrostar Nano, BMG Labtech, Ortenberg, Germany).

### 2.12. Analysis of mRNA and miRNA Expression

After 72 h the cultures were homogenized using TRI reagent. Total RNA was isolated accordingly to the method described previously by Chomczynski and Sacchi [26]. The obtained total RNA was diluted with molecular-grade water. The quantity and quality of specimens were determined using a spectrophotometer (WPA Biowave II, Cambridge, UK). Genomic DNA (gDNA) was removed using a DNase I RNase-free kit (Thermo Fisher Scientific, Warszawa, Poland). The reaction was performed according to the guidance of the producer.

The synthesis of cDNA for the detection of target genes (Bax, Bcl-2, FOXO, p53, and p21) was performed using a Tetro cDNA Synthesis Kit (Bioline, London, UK). Each reaction contained 500 ng of totalRNA. Quantitative RT-PCR was carried out in a total volume of 20 µL using a SensiFast SYBR and fluorescein Kit (Bioline, London, UK). For the reaction, the following cycling conditions were applied: 95 °C for 2 min, followed by 60 cycles at 95 °C for 15 s, annealing for 15 s, and elongation at 72 °C for 15 s with a single fluorescence measurement.

To determine miRNA expression, 375 ng of RNA was reverse-transcribed using a Mir-X miRNA First-Strand Synthesis Kit (Takara Bio Europe, Saint-Germain-en-Laye, France). The obtained matrices were used for quantitative PCR (final volume 20 µL) with SYBR Advantage qPCR Premix (also derived from Takara Bio Europe). The reaction included the initial denaturation at 95 °C for 10 s, followed by 55 cycles of 95 °C for 5 s and annealing temperature 60 °C for 20 s with a single fluorescence measurement.

All reactions were performed in three repetitions. Specificity of the PCR products was determined by the analysis of the dissociation curve of amplicons. The melting curve was performed using a gradient program in the range from 65 to 95 °C at a heating rate of 0.2 °C/s and with continuous measurement of the fluorescence. Additionally, the miRNA products were analyzed by electrophoresis in 2% agarose gel (Novazym, Poznan, Poland) stained with ethidium bromide (Sigma Aldrich, Munich, Germany). The average fold change in the gene expression of experimental cultures was compared with control cultures and calculated by the 2^−DDCt^ method in relation to the housekeeping gene—GAPDH for target genes, and U6snRNA for miRNA. Sequences of primers used for detection mRNA are listed in Table 1, while for miRNA are listed in Table 2.

### 2.13. Statistical Analysis

All experiments were performed in triplicate. The analysis of data obtained in biological assays were analyzed with STATISTICA 10.0 software (StatSoft, Inc., Statistica for Windows, Tulsa, OK, USA). The normality of the population data was determined using the Shapiro-Wilk test, while equality of variances was assessed by Levene’s test. Differences between groups were determined using one- or two-way analysis of variance (ANOVA). Differences with a probability of *p* < 0.05 were considered as significant.

## 3. Results

### 3.1. Spirulina Reduces the Size of Caco-2 Colonies and May Alter Proliferation Activity of Caco-2

**Figure 1:**
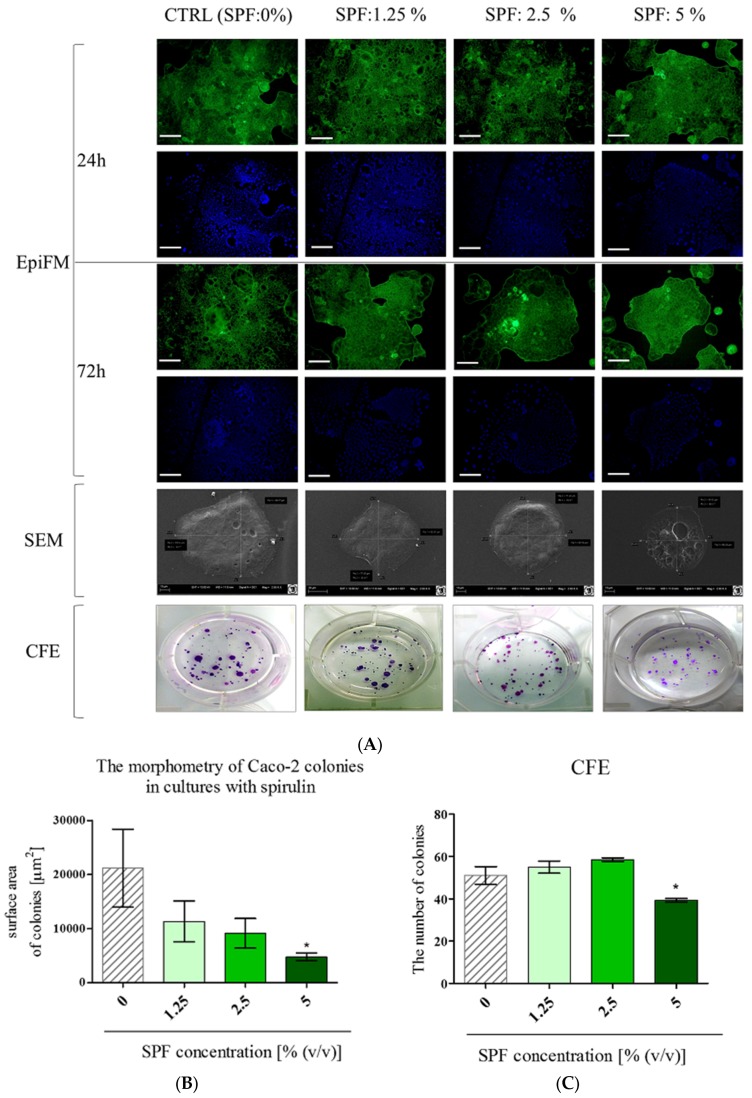

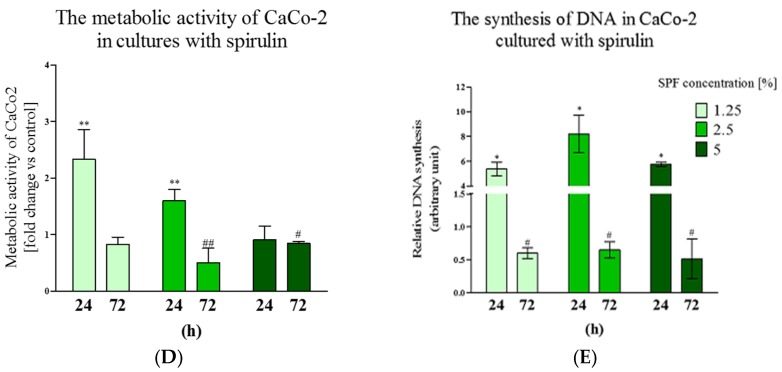
The effect of *Spirulina platensis* filtrates on culture morphology and proliferative activity. (**A**) The Caco-2 cells were captured using an epifluorescence microscope (EpiFM; scale bar = 250 μm) after 24 and 72 h of culture. The decline of Caco-2 colony sizes was observed (SEM; scale bar = 20 μm), however, this was associated with a colony-forming efficiency increase. The decrease of Caco-2 colony surface areas (**B**) and numbers (**C**) was observed in cultures with SPF at 5%. The SPF addition altered Caco-2 metabolic (**D**) and proliferative (**E**) activity—the effect dependent on culture duration and SPF concentration. Statistically significant differences were indicated with an asterisk (*) or hashtag (#), in order to distinguish, respectively, the improvement or decrease of investigated feature. The statistically significant differences were determined at *p* < 0.05 (* or #); *p* < 0.01 (** or ##).

We examined the effect of SPF on the morphology and proliferation activity of Caco-2 cells, after 24 and 72 h of culture (Figure 1). Evident changes in Caco-2 colonies were notable after 72 h. The addition of SPF influenced colony sizes. The Caco-2 cultures are characterized by enterocyte-like phenotype and form large and pseudostratified colonies. In cultures with *Spirulina* Caco-2 created multiple spherical colonies, however, more compact and reduced in size. The morphometry analysis showed that the size of colonies decreased in a dose-depended manner, however, a statistically significant decrease of surface area of Caco-2 colonies was noted only in cultures with SPF at a concentration 5% (*v*/*v*) (Figure 1B). The clonogenic potential of Caco-2 cultures was also affected by SPF—the number of colonies slightly increased in cultures treated with SPF at 1.25% and 2.5%—but the difference was not statistically significant. SPF at 5% significantly lowered the number of CFU in comparison to the control cultures (Figure 1C).

The analysis of metabolic and proliferative status of Caco-2 showed that SPF addition increased their activity after 24 h; just metabolic activity of cultures with 5% of SPF remained unaffected. The analysis performed at 72 h, showed that the decrease of metabolic activity at a statistically significant level was observed in cultures with SPF at concentrations 2.5% and 5%, while the lowered proliferation ratio was noted in Caco-2 in all cultures with SPF (Figure 1D,E).

### 3.2. High Concentration of Spirulina May Inhibit Caco-2 Migration

**Figure 2:**
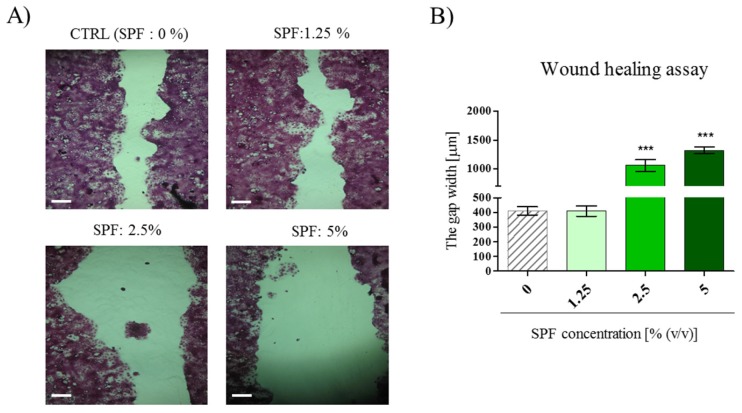

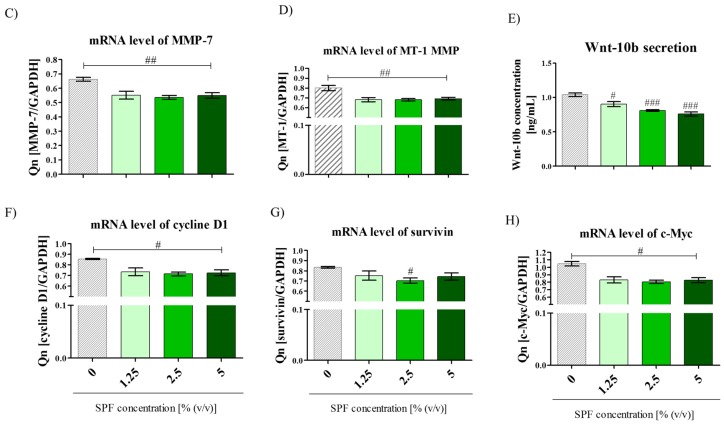
The effect of SP on the migration of Caco-2 cells. (**A**) The images of wound site in Caco-2 cultures with and without SPF (scale bar = 250 μm); (**B**) the gap width was measured and represented as mean ± SD of quadruplicate cultures from triplicate experiments, the increase in a wound gap was significant *p* < 0.001; (**C**,**D**) the transcript levels of MMP-7 and MT-1 after SPF treatment; (**E**) the effect of SPF on Wnt-10b autocrine secretion determined with the ELISA; (**F**–**H**) the mRNA levels of downstream target genes of the signaling pathway crucial for tumor survival. Statistically significant differences were indicated with hashtag (#) in order to mark the decrease of investigated feature. The statistically significant differences were noted at *p* < 0.05 (#); *p* < 0.01 (##) and *p* < 0.001 (###).

To determine the effects of SPF on the migration capability of Caco-2 cells, the wound healing assay was performed on confluent monolayers. Obtained results indicated that migration of Caco-2 was not affected by SPF at a concentration of 1.25%, however SPF at concentrations of 2.5% and 5% evidently inhibited the migratory capability of Caco-2 by 60%–70% (Figure 2A,B). This is consistent with the analysis of mRNA expression of matrix metalloproteinases, which are crucial for cell migration. We observed the decrease in transcript levels for MMP-7 and MT-1 following SPF treatment (Figure 2C,D). Additionally, the SPF negatively influenced the secretion of Wnt10b in Caco-2 cultures. Wnt10b autocrine secretion was significantly decreased in cultures with SPF. This was accompanied by significantly lowered mRNA expression for cyclin D1, surviving, and c-Myc (Figure 2E–H).

### 3.3. Spirulina Disturbs on Mitochondrial Activity in Caco-2 and Increases Bax/Bcl-2 Ratio

**Figure 3:**
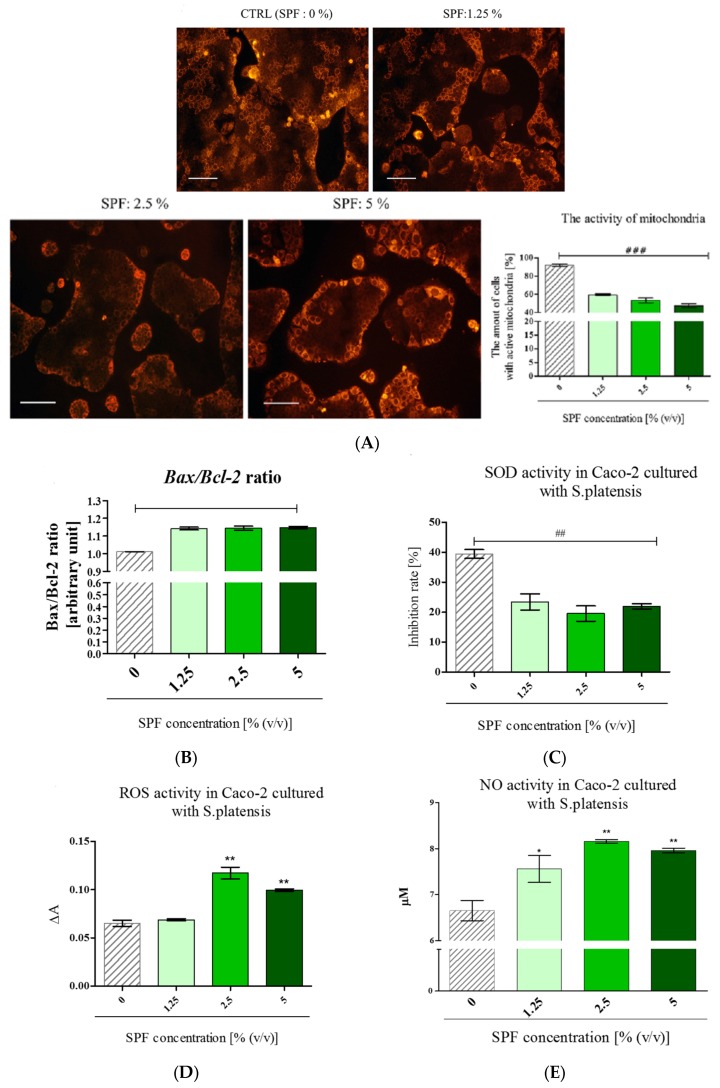
The influence of *S. platensis* on mitochondrial activity, Bax/Bcl-2 ratio, and the level of oxidative stress markers. (**A**) The visualization and analysis of cultures after MitoRed staining (scale bar = 250 μm); (**B**) transcript level of pro-apoptotic Bax and anti-apoptotic Bcl-2 expressed as a ratio; the activity of superoxide dismutase activity (**C**), reactive oxygen species (**D**) and nitric oxide (**E**). Statistically significant differences were indicated with an asterisk (*) or hashtag (#) in order to distinguish, respectively, the improvement or decrease of investigated feature. The statistically significant differences were tested at *p* < 0.05 (* or #); *p* < 0.01 (** or ##); *p* < 0.001 (*** or ###).

Mitochondria in Caco-2 cultures were visualized using fluorescent Mito Red staining. The analysis of staining intensity showed that the number of active mitochondria in Caco-2 decreased in cultures with SPF in a dose-dependent manner. Additionally, the intense fluorescence signal of Mito Red was observed mainly in cells forming outside layers of colonies (Figure 3A). In addition, following the SPF treatment the Bax/Bcl-2 ratio increased (Figure 3B). Furthermore, to determine the possible mechanism of SPF cytotoxic action the oxidative stress markers were evaluated. The analysis revealed that SOD activity was significantly lowered in cultures with SPF addition. The SOD level in all cultures with SPF decreased by approximately 50% when compared to non-treated cultures (Figure 3C). Simultaneously, the increased ROS and NO activity was noted in cultures with SPF at concentrations of 2.5% and 5% (Figure 3D,E). This result suggests that intracellular ROS and NO generated in Caco-2 cultures after SPF treatment may be involved in the induction of cell death.

### 3.4. The Percentage of PI Stained Cells Increased in Caco-2 Cultures with S. platenis in a Dose-Dependent Manner

**Figure 4:**
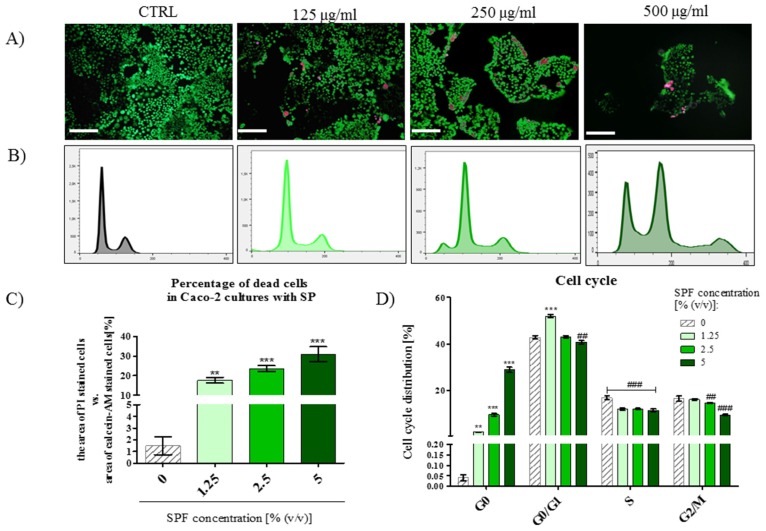
The results of live/dead staining and cell cycle progression examined by flow cytometry. (**A**) The images presenting viability of cells (scale bar = 250 μm), live cells are stained with calcein (green signal), while dead are propidium iodide positive (purple signal); (**B**) the histograms presenting cell cycle distribution; (**C**) the results of propidium iodide positive cells determination; (**D**) the statistical analysis of cells cycle distribution. Statistically significant differences were indicated with an asterisk (*) or hashtag (#) in order to distinguish, respectively, the improvement or decrease of investigated feature. The statistically significant differences were tested at *p* < 0.05 (* or #); *p* < 0.01 (** or ##); and *p* < 0.001 (*** or ###).

Cell death induced by SPF was determined by the live/dead assay (Figure 4A), while the cell cycle was analyzed by flow cytometry (Figure 4B). Due to the differences in the size of Caco-2 colonies, the percentage of dead cells, stained with propidium iodide, was evaluated in regards to the area occupied by living cells, visualized with calcein-AM staining (Figure 4C). The percentage of dead cells increased along with the increasing SPF concentration, thus obtained results are another confirmation of dose-dependent cytotoxicity of SPF against Caco-2 cell line. Moreover, the analysis of cell cycle distribution confirmed that Caco-2 treated with SPF underwent cell death, as judged by the increase on sub-G0 percentage. The percentage of proliferating cells (S phase) was also significantly lowered after SPF treatment (Figure 4B,D).

**Figure 5:**
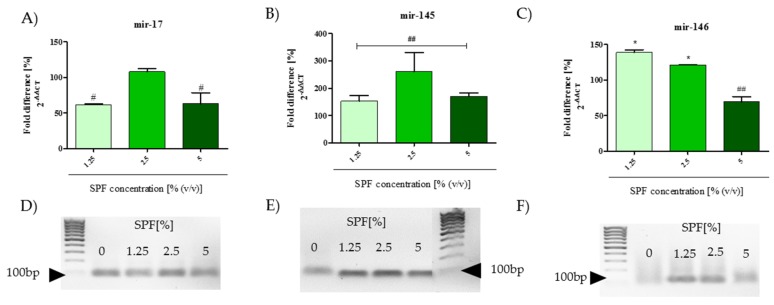
The expression of miRNAs associated in tumorgenesis of Caco-2. (**A**) The transcript level of mir-17, (**B**) mir-145 and (**C**) mir-146; (**D**–**F**) electrophoregrams of qPCR products for mir-17, -145 and 146, respectively. Statistically significant differences were indicated with an asterisk (*) or hashtag (#) in order to distinguish, respectively, the improvement or decrease of investigated feature. The statistically significant differences were tested at *p* < 0.05 (* or #); *p* < 0.01 (** or ##).

Additionally, the expression of miRNA17 was downregulated or remained unchanged in response to SPF treatment (Figure 5A,D). Mir-145 expression was noted in all tested cultures in regard to control conditions (Figure 5B,E). The significant upregulation of the miR-146 transcript level was noted in cultures with 1.25% and 2.5% of SPF. However, the highest concentration of SPF decreased the miR-146 level in Caco-2 (Figure 5C,F). The obtained results indicate the loss of invasive ability of Caco-2 and are in good agreement with results obtained in the wound-healing assay.

## 4. Discussion

Colorectal cancer is one of the most common malignancies, and the incidence of this disease is steadily increasing [27]. The GLOBOCAN statistics showed 1.4 million new cases that were diagnosed in 2012 [27,28,29]. The overall five-year survival rate was estimated at 90% in patients with the localized stage of the disease. However, in patients with distant metastases the survival rate is only 5%–8% [28]. These statistics show that colorectal cancer is a serious clinical problem worldwide [30]. The higher risk of colon cancer occurrence is linked with unhealthy dietary habits, i.e., increased consumption of saturated and hydrogenated fats, and high-energy foods [29,30]. There is a great emphasis on the role of proper nutrition in preventing colon cancer incidence. Mounting evidence indicates that algae-based food is an essential source of anti-cancer and anti-inflammatory phytochemicals, which may find application as agents preventing colorectal tumorigenesis [29,31,32].

One of the main aspects of the present study was to investigate the influence of *Spirulina platensis* filtrates (SPF) on proliferative activity of a human colon cancer cell line Caco-2. The anti-cancer activity of *Spirulina* was explained by its anti-proliferative effect observed on various cancer cell lines. The bioactivities found in *Spirulina*, for example, c-phycocianin and tetrapyrrolic compounds, exerted anti-proliferative effects in vitro on a myeloid leukemia cell line [33], but also on pancreatic cancer cell lines [17] and a hepatocellular carcinoma cell line [11]. Nonetheless, its influence on human colon cancer cell lines seems to be less thoroughly investigated [29]. In the present study, we showed that SPF had an ambiguous effect on the metabolic and proliferative activity of Caco-2. The proliferation of Caco-2 cultures treated with SPF for 24 h was significantly accelerated. Additionally, SPF at concentrations equal 1.25% and 2.5% (*v*/*v*) enhanced the metabolic status of Caco-2. In turn, after 72 h of Caco-2 propagation with SPF, both metabolic and proliferative activity was lowered. The similar response on super-cocktail of known phytochemicals was determined for a metastatic breast cancer cell line, i.e., MDA-MB-231—the cytotoxic effect was observed by day three of treatment, indicating on increased resistance to therapy during a short period [34]. The inhibition of Caco-2 proliferation observed at 72 h with SPF could result from the reduction of the cell numbers due to the induced cell death, or could be caused by the inhibition of the cell cycle. The analysis of cultures’ morphology performed in this study provided the first answers regarding this dilemma. The morphology of Caco-2 was monitored, both after 24 and 72 h of cultures with SPF, and the results were consistent with the analysis of the Caco-2 growth rate. No significant alterations of cultures’ morphology were observed after 24 h treatment with SPF, however, after 72 h we observed shrinking of Caco-2 colonies. The most evident changes in Caco-2 morphology, reflecting the hallmarks of cell apoptosis, were noticed at the highest concentration, i.e., 5% (*v*/*v*). Our observations are in good agreement with previous studies showing the cytotoxic effects of macroalgal extracts induced in breast cancer cell lines, and the series of morphological changes related to apoptosis, including cell shrinkage [35,36].

Furthermore, we were interested if the decreased growth rate of Caco-2, coupled with reduced colony size after SPF treatment, may impact clonogenic potential of Caco-2. In the present study, the clonogenic assay allowed the evaluation of the long-term cytotoxic effect of SPF on Caco-2 cultures. Again, the highest concentration of SPF showed the cytotoxic effect and significantly reduced the number of Caco-2 colonies, which supports previous results. Coordinate proliferation and migration of cells reflects the metastatic potential of cells, and can be measured using a wound-healing assay. The results of our experiment showed that anti-proliferative and anti-clonogenic properties of the highest concentration of SPF also influenced Caco-2 migratory capabilities. These observations correspond with the results of Han et al. who showed that fucoidan, derived from brown algae, inhibited migration of human colon cancer cell line HT-29 [37]. Another bioactive component isolated from brown algae—fucoxanthin—was shown as an agent that markedly suppresses cell migration in wound healing assays [38]. Both studies highlighted the fact that algal derivatives with anti-cancer properties may affect the expression of matrix metalloproteinases (MMP) involved in extracellular matrix (ECM) degradation. The proteolytic activity of metastatic tumor cells is their intrinsic feature, allowing them to transmigrate through the basal membrane and further, with invasion through the stroma [39]. Colorectal tumor progression was associated with overexpression of MMP-7 [40] and MMP-14, which is also known as a membrane type 1-matrix metalloproteinase (MT1-MMP) [39,41]. In the present paper we showed that SPF may decrease mRNA expression of MMP-7 and MT1-MMP. Previously mentioned, Han et al. [37] and Chung et al. [38] noted that MMP expression in colon cell lines decreased in a dose-dependent manner after treatment with brown-algae bioactivities. Here, we observed that MMP-7 and MT-MMP mRNA levels decreased significantly, regardless of the used SPF concentrations. However, the dose-dependent mechanism of SPF action was observed in relation to the Wnt-10 protein secretion. The Wnt signaling activity is required in colon cancer cells during tumor progression and metastasis [42], thus, inhibition of Wnt-10 secretion observed in Caco-2 propagated with SPF may explain the results obtained in the wound-healing assay. Additionally, autocrine action of secreted Wnt has an important role in colon cancer development, not only regulating proliferation, but also maintaining survival of colon cancer cells [43]. In the present study, we decided to determine the mRNA expression of Wnt target genes, such as anti-apoptotic survivin (BIRC5), cell-cycle regulators, namely cyclin D1 and c-myc. A number of earlier studies indicate that higher expression of these genes occurs in various cancers, including colorectal cancer [44,45]. In the present study we showed that the inhibition of growth and migration in Caco-2 cultures treated with SPF may be associated with downregulation of pro-survival genes. The same mechanism of action was described for fucoidan implemented to the 4T1 mouse breast cancer cell line [37] and quercetin tested on the human SW480 colon cancer cell line [46].

Mounting evidence indicates that bioactive compounds extracted from algae exerted anti-cancer effects through multiple mechanisms of action. The inhibition of cancer cell growth and migration is one of them, and this was also supported by our results, but anti-cancer effects may be also triggered with the induction of apoptosis in cancer cells [29]. In this paper, we showed that the apoptotic pathway in Caco-2 cells treated with SPF may occur via modulation of Bax and Bcl-2 expression and may be associated with the disruption of the mitochondrial membrane potential. It is well known, that due to antagonistic action of Bax and Bcl-2, it is possible to determine their ratio, related to the sensitivity or resistance of the cells to various apoptotic stimuli. While pro-apoptotic Bax promotes the release of cytochrome C into the cytosol from mitochondria, the anti-apoptotic Bcl-2 preserves the integrity of the mitochondria [29,47,48,49]. Our results indicated that SPF treatment in Caco-2 increased the Bax/Bcl-2 ratio and lowered mitochondrial activity (Mito Red staining). This observation, along with downregulation of survivin mRNA expression, implies that molecular events occurring during mitochondria-mediated apoptosis were activated in Caco-2 as a response to the SPF stimuli. Moreover, the morphological changes indicative of apoptosis, were also evaluated after calcein-AM and iodide propidium staining, and indicated that SPF not only reduced the size of colonies, but also caused accumulation of non-viable cells. In addition, following the SPF treatment, an increase in the population of apoptotic sub-G0 phase cells was noted. Furthermore, investigation of the possible mechanism underlying the induction of apoptosis, observed Caco-2 death after SPF treatment may be associated with oxidative stress. In the current research, we have found that activity of superoxide dismutase in Caco-2 was reduced following SPF treatment, in parallel with elevated reactive oxygen species and nitric oxide levels. Such a profile of oxidative stress markers, i.e., with low levels of SOD and elevated levels of ROS/NO, is associated with depolarization of the mitochondrial membrane, which may regulate apoptotic signal transduction. It seems that the exact influence of algal compounds and their metabolites on oxidative status in cancer cell lines is not clear. Mounting evidence indicates that antioxidant compounds are present in the extracts of *S. platensis* [50]. For example Konickova et al. [18] showed that anticancer effects of tetrapyrroles derived from *S. platensis* are associated with direct inhibition of mitochondrial ROS generation. Similarly, another microalga—*Pediastrum duplex*—was shown to be an excellent source of naturally-occurring antioxidant compounds with a protective role against DNA damage induced by H_2_O_2_ in a lymphoma cell line [51]. Simultaneously, it was shown that ethyl acetate extracts derived from three particular algae (i.e., *Colpomenia sinuosa, Halimeda discoidae*, and *Galaxaura oblongata*) induced apoptosis in human leukemia cells through the generation of ROS [52]. The same mechanism of action was observed toward rat histiocytic tumor line AK-5, namely C-phycocyanin derived from *Spirulina platensis* induced apoptosis through the production of ROS, which was suppressed by Bcl-2 [53]. In our opinion, our results strongly correspond with the study of Ryu et al. [48], who showed that a green alga *Ulva fasciata* induced ROS-mediated apoptosis in HCT 116 human colon cancer cells, and this process may be associated with decreased Bcl-2 level and enhanced expression of Bax. The recent findings show that induction of oxidative stress appears to be a promising approach for the preferential killing of cancer cells, including CSC [54]. This sheds a new light on the application of algae and its major active components in anticancer therapies as modulators of redox status that may selectively target cancer cells, without affecting normal cells.

The oxidative stress may also alter the expression of non-coding micro RNAs (miRNAs) [55]. The most current studies are focused on the analysis of the miRNAs involvement in the processes of oncogenesis, also in the course of colon cancer [56]. In the present study we investigated the expression of miR-17, miR-145 and miR-146 identified as biomarkers of colon cancer [57,58]. The obtained results indicated that SPF may modulate the expression of miRNA, the most significant changes in the miRNAs expression profile were noted in cultures with the highest concentration of SPF. The expression of miR-17 and miR-146 was downregulated, while the expression of miR-145 was upregulated. This is consistent with the role of these miRNAs in cancer development—the overexpression of miR-17 and mir-146 is a feature of hyper-proliferating cancer cells [20,59], while the enhanced expression of miR-145 impairs proliferation of the HCT116 colon cancer cell line [60].

## 5. Conclusions

Based on the results, we concluded several critical links which might have influence on apoptosis of colon cancer cell line Caco-2 treated with *Spirulina platensis* filtrates. The SPF inhibited growth and migration of the Caco-2 cell line by modulating the expression of MMP-7 and apoptosis-related genes. The SPF enhanced the expression of pro-apoptotic and down-regulated expression of anti-apoptotic genes, which might be associated with ROS-induced oxidative stress. To the best of our knowledge this is the first report demonstrating the cytotoxic effect of SPF using the Caco-2 model. Presented results, supported by the in vivo studies performed by other research groups, shed promising light for the application of *S.platensis* in colon cancer prevention and treatment.

## Figures and Tables

**Table 1:** Sequence of primers used for detection of chosen genes.

Gene	Primer	Sequence 5′-3′	Loci	Ta [°C]	Amplicon Length [bp]	Accesion No.
*Bax*	F:	ACCAAGAAGCTGAGCGAGTGTC	235–256	59.6	365	NM_001291428.1
	R:	ACAAAGATGGTCACGGTCTGCC	627–648			
*Bcl-2*	F:	ATCGCCCTGTGGATGACTGAG	1010–1030	58.6	129	NM_000633.2
	R:	CAGCCAGGAGAAATCAAACAGAGG	1115–1138			
*p21*	F:	AGAAGAGGCTGGTGGCTATTT	21–41	57.9	169	NM_001220777.1
	R:	CCCGCCATTAGCGCATCAC	171–189			
*p53*	F:	AGATAGCGATGGTCTGGC	868–885	57.8	381	NM_001126118.1
	R:	TTGGGCAGTGCTCGCTTAGT	1229–1248			
*c-Myc*	F:	CTTCTCTCCGTCCTCGGATTCT	1215–1236	58.2	204	NM_002467.4
	R:	GAAGGTGATCCAGACTCTGACCTT	1395–1418			
*cyclinD1*	F:	GATGCCAACCTCCTCAACGA	264–283	58.2	211	NM_053056.2
	R:	GGAAGCGGTCCAGGTAGTTC	455–474			
*survivin*	F:	ACCGCATCTCTACATTCAAG	171–190	58.2	113	NM_001012271.1
	R:	CAAGTCTGGCTCGTTCTC	266–283			
*MMP7*	F:	TGTATGGGGAACTGCTGACA	494–513	58.2	151	NM_002423.4
	R:	GCGTTCATCCTCATCGAAGT	625–644			
*MT1-MMP*	F:	TCGGCCCAAAGCAGCAGCTTC	364–384	58.2	180	NM_004995.3
	R:	CTTCATGGTGTCTGCATCAGC	523–543			
*GAPDH*	F:	GTCAGTGGTGGACCTGACCT	894–913	59.1	256	NM_001289746.1
	R:	CACCACCCTGTTGCTGTAGC	1130–1149			

**Table 2:** Sequences of primers used for detection of microRNA.

Gene	Primer	Sequence 5′-3′	Location	Ta [°C]	Amplicon Length [bp]	Accesion No.
Hsa-miR-17-5p	F:	CAAAGTGCTTACAGTGCAGGTAG	13q31.3	57.4	*	MIMAT0000070
Hsa-miR-145-5p	F:	GTCCAGTTTTCCCAGGAATCCCT	5q32	58.8	*	MIMAT0000437
Hsa-miR-146a-5p	F:	TGAGAACTGAATTCCATGGGTT	5q33.3	61	*	MIMAT0000449
U6	F:	GCTTCGGCAGCACATATACTAAAAT	16q21	60	*	NR_125730.1
	R:	CGCTTCACGAATTTGCGTGTCAT				

* Due to poly (A) sequence it is not possible to predict the actual product size.
